# A Large Pelvic Haematoma Caused by a Local Anaesthetic Flexible Cystoscopy Injection of Intradetrusor Botulinum Toxin A: A Case Report

**DOI:** 10.7759/cureus.74754

**Published:** 2024-11-29

**Authors:** Andrew Atayi, Saleh Al-Gburi, Musaab Hamdoon, Nina Patrick

**Affiliations:** 1 Urology, Wirral University Teaching Hospital NHS Foundation Trust, Wirral, GBR

**Keywords:** botulinum toxin, cystoscope, overactive bladder, pelvic haematoma, urology

## Abstract

A 55-year-old female attended the Outpatient Urology Department for local anaesthetic flexible cystoscopy and intradetrusor botulinum toxin A injection. Having been diagnosed with urodynamics-proven low-grade detrusor overactivity in 2017, she was well-established on six-monthly Botox® injections. As part of her ongoing treatment, 100 units of Allergan Botox diluted with saline in a 10 mL syringe were injected via 20 punctures. There were no immediate complications. Three days later, she presented with abdominal pain and haemorrhagic discolouration around her umbilicus (Cullen’s sign). Haemoglobin had dropped by 37 g/L, from 130 to 93 g/L. An urgent CT of the abdomen and pelvis with contrast revealed a large 10 x 4 cm pelvic haematoma, which was managed conservatively. A repeat CT of the abdomen and pelvis with contrast three weeks later showed no change in the pelvic haematoma size, but a reduction in attenuation suggested partial liquefaction.

## Introduction

Bladder botulinum toxin administration is indicated in patients with idiopathic overactive bladder (OAB) syndrome and neurogenic detrusor overactivity (DO) [1]. Botulinum toxin is a neurotoxin produced by the anaerobic gram-positive bacterium *Clostridium botulinum*. Subtypes A, B, E and F are known to cause botulism in humans. Botulinum toxin A is commonly used in therapeutic doses under a variety of preparations - Botox®, Dysport® and Xeomin® [2]. It is administered as an intradetrusor injection via rigid or flexible cystoscopy. In most centres, this is usually under local anaesthetic; however, a select few patients will undergo a general anaesthetic. Side effects from botulinum toxin A are usually mild and self-limiting, including haematuria, uncomplicated urinary tract infection and urinary retention requiring intermittent or temporary indwelling catheterisation. Rare complications such as allergic reactions and generalised muscle weakness can occur in less than 0.4% of patients [3]. We report an unusual case of a large pelvic haematoma caused by a local anaesthetic flexible cystoscopy injection of intradetrusor botulinum toxin A.

## Case presentation

A 55-year-old female patient attended for her six-monthly intradetrusor botulinum toxin A injections via flexible cystoscopy under local anaesthetic. She was not established on anti-coagulant or anti-platelet medication. Pre-procedure urine dip was negative for erythrocytes, leucocytes and nitrites. The procedure was performed without immediate complication by a specialist urologist. As part of her ongoing treatment, 100 units of Allergan Botox were injected via 20 different sites, avoiding the trigone using a 120 cm, 18-gauge needle (MedProvisions, China) measuring 4 mm at the needle tip.

**Figure 1:**
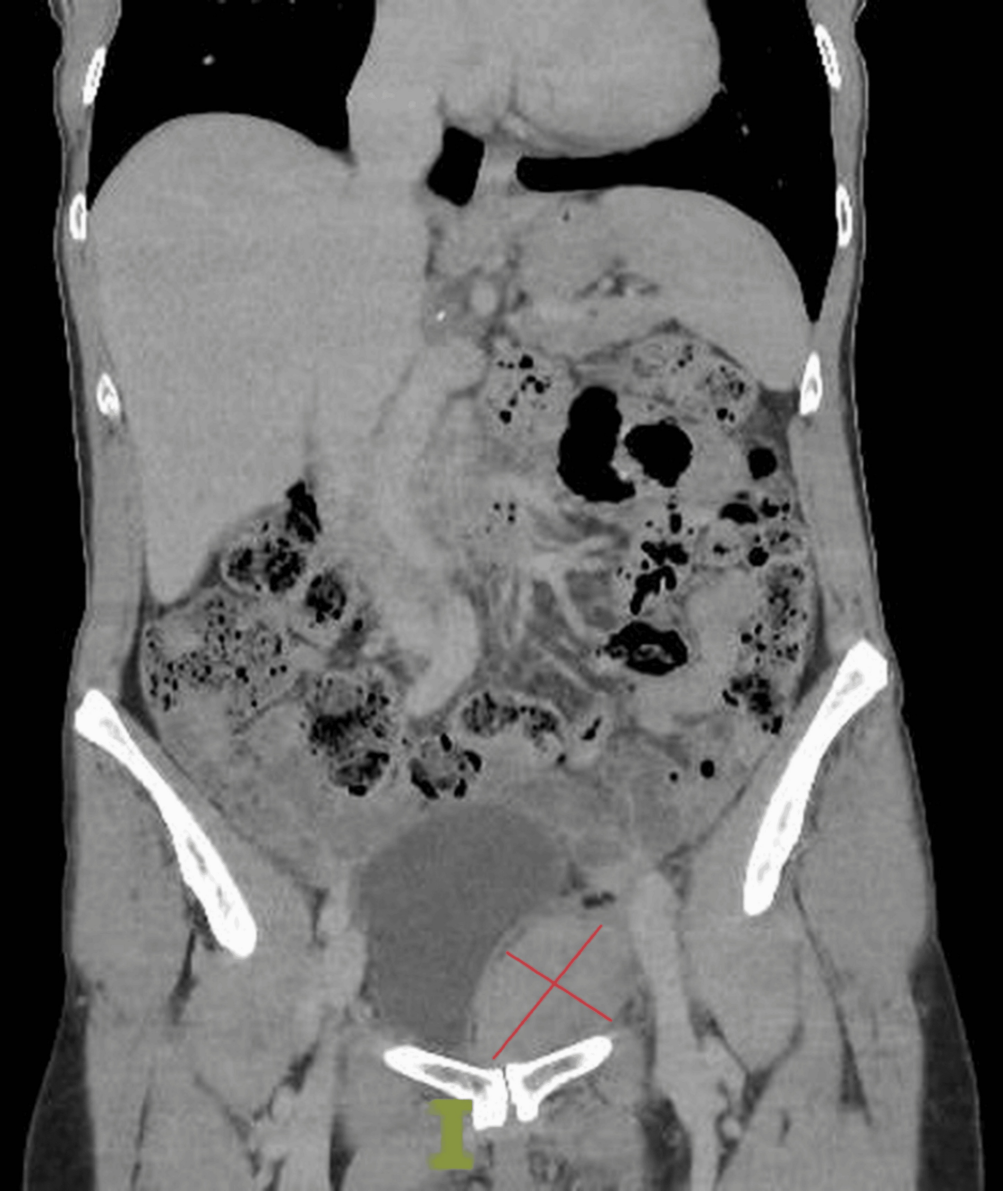
Coronal spiral CT scan with arterial and portovenous contrast phases showing 10 x 4 cm left pelvic haematoma

The patient presented acutely to the urology ‘hot clinic’ three days later with lethargy and abdominal pain. She was haemodynamically stable and had no visible haematuria. Examination revealed mild peri-umbilical bruising tracking down towards the suprapubic region. Laboratory blood tests were obtained. Full blood count showed a haemoglobin of 93 g/L (112-148 g/L), previously 130 g/L pre-procedure. Haematocrit was 0.26 (0.34-0.45). Platelets were 210 x 10^9/L (150-400 x 10^9/L) and INR was 0.9. Urea and creatinine were 6.4 mmol/L (2.5-7.8 mmol/L) and 74 mmol/L (45-84 mmol/L) respectively. She was admitted for an urgent spiral CT scan with arterial and portovenous contrast phases which showed a large 10 x 4 cm high-intensity collection within the left pelvis consistent with a retroperitoneal haematoma which was managed conservatively (Figure 1).

**Figure 2:**
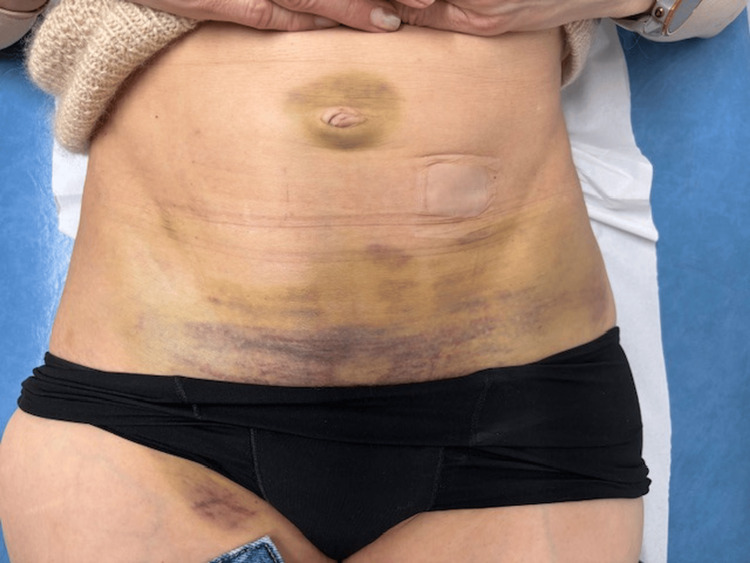
Resolving superficial bruising, previously tracking from periumbilicus to suprapubic region

She was discharged the following day, given she was clinically well. When reviewed in an outpatient clinic one week later, she reported symptomatic improvement. Repeat blood tests showed haemoglobin had improved to 110 g/L. Examination showed gradual resolution of the superficial bruising, with yellowing (haemoglobin degradation) at the edges (Figure 2).

**Figure 3:**
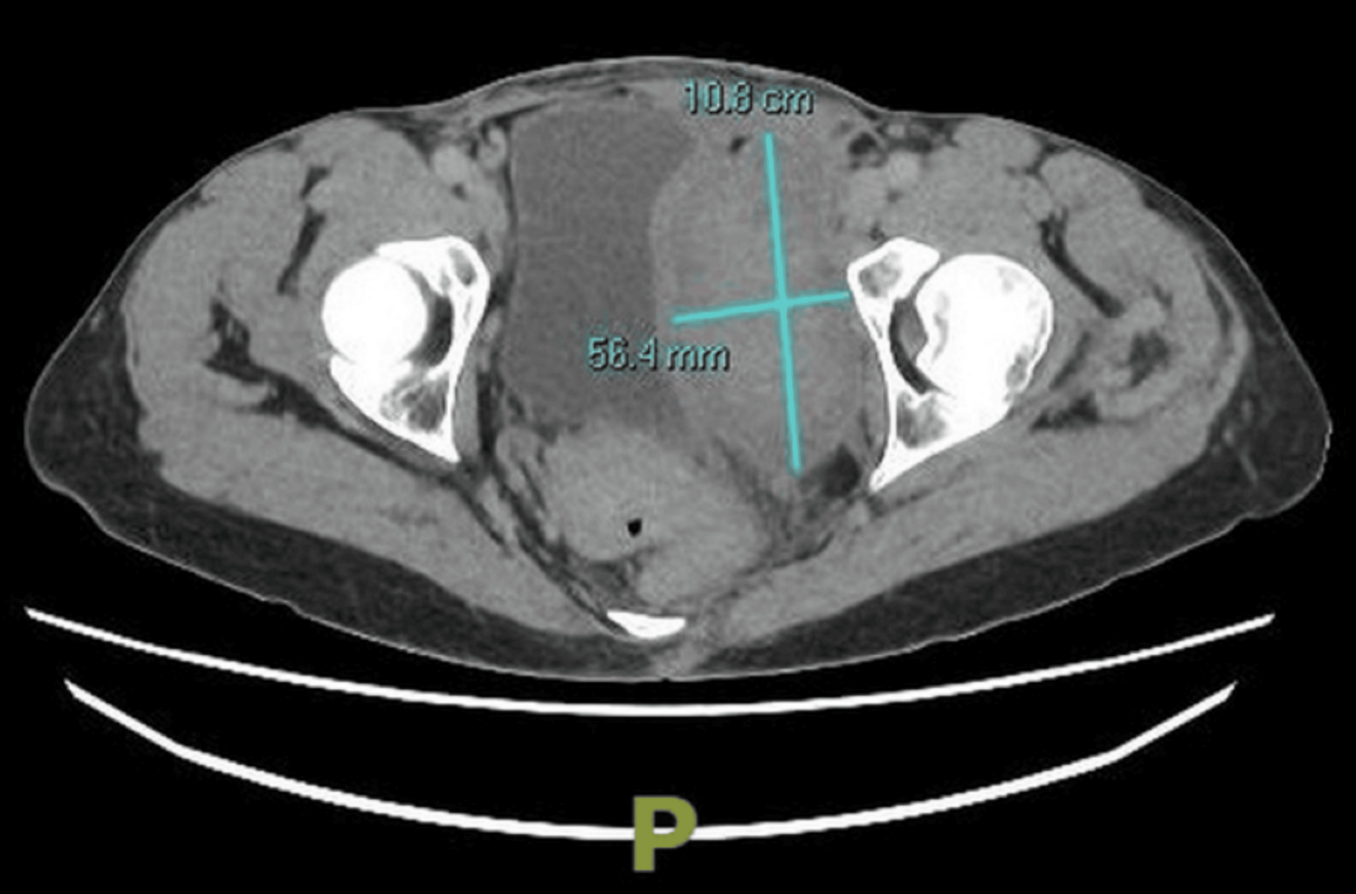
Axial spiral CT scan with arterial and portovenous contrast phases showing reduction in attenuation indicating resolution

A repeat contrast CT scan of the abdomen and pelvis, three weeks later, showed no significant change in pelvic haematoma size, but a reduction in attenuation suggesting partial liquefaction (Figure 3).

## Discussion

OAB is associated with four main symptoms: urinary urgency, urge incontinence, frequency and nocturia. DO is characterised physiologically by involuntary detrusor contractions that occur spontaneously or in response to stimulation [3]. It is presumed idiopathic in over 90% of women who present with symptoms. Seldom is an identifiable neurological condition found [4]. However, multiple sclerosis, dementia, Parkinson's disease, spinal cord injuries, cerebrovascular accidents and other neurologic disorders are frequently linked to neurogenic DO [5].

Karsenty et al. found most issues from Botox were injection site pain, procedure-related urinary tract infection (2%-32%), mild haematuria (2%-21%) and an increase in post-void residual (PVR) volume occasionally leading to intermittent self-catheterisation (6%-88%) or urinary retention (0%-33%) [6].

Botulinum toxin injections into the lower urinary tract rarely cause systemic consequences. However, there are theoretical concerns about systemic consequences because of botulinum toxin's paralytic action. Blurred vision, diplopia, dysphagia and generalised weakness are possible adverse effects [7].

To the best of our knowledge, pelvic haematoma following intradetrusor botulinum toxin A has never been documented in the literature. Although haematuria lasting one to three days is to be expected in ‘almost all cases’ as per the British Association of Urological Surgeons (BAUS) consent leaflet, extra/intraperitoneal bleeding is not a recognised theoretical or actual complication [8]. Interestingly, in this case, the patient did not report any visible haematuria. Normal bladder wall thickness in adult women is 3 ± 1 mm. This is known to decrease with age and distension of the bladder [9]. A distended female bladder can be paper thin and hence presents a considerable risk of perforation during bladder biopsy or transurethral resection. We postulate that the Botox needle passed in its entirety through the layers of the bladder and inadvertently punctured a branch of the superior vesical artery or a branch of the vaginal artery supplying the bladder, along the lateral aspect. Injury to the vesical venous plexus could also be possible, although one would not expect such a significant haematoma to form.

The location and number of injections given is a contentious issue with no universally agreed evidence-based protocol. Neither the National Institute for Health and Care Excellence (NICE) nor the European Association of Urology stipulate on the process of administering Botox; however, no more than 200 units of Botox should be given in one setting. The safe number of units is different for Dysport and Xeomin. Most clinicians will aim to give 10-20 injections evenly around the bladder whilst avoiding the trigone due to the risk of causing vesico-ureteric reflux. There is limited evidence as to whether reducing the number of injections reduces the likelihood of a painful experience or bleeding [10]. Interestingly, in this case, the patient had previously had 10 injections with no reported complications.

## Conclusions

Intradetrusor botulinum toxin A is a well-established, safe procedure with few significant risks. However, care must be taken, particularly in the female bladder, to avoid extra-vesical vascular injury. Ensuring the bladder is not overdistended, minimising the number of injections and using the correctly sized Botox needle may reduce the risk of this rare complication.
